# Establishing an ethics and governance framework for access to and linkage of electronic health data for research projects.

**DOI:** 10.23889/ijpds.v7i3.1958

**Published:** 2022-08-25

**Authors:** Emily Parker, Tanya Ravipati, Richard Beare, Velandai Srikanth, Nadine Andrew

**Affiliations:** 1 National Centre for Healthy Ageing

## Objectives

To develop an ethics and governance framework for the National Centre for Healthy Ageing (NCHA) data platform that supports: streamlined access to data for research; transfer of data into secure data environments; linkage with a range of external data sources and incorporation of a variety of data types.

## Approach

The NCHA data platform is bringing together Electronic Health Data across an entire region for health service and clinical research. Methods used to establish the framework include: review of existing national (Australian Institute of Health and Welfare’s data governance framework) and international (Guiding principles from ISO/IEC 385051:2017) frameworks, stakeholder engagement and early piloting through use cases. End-users and executive staff (clinical, research and legal) were consulted to ensure compliance and streamlining with existing processes. A data access governance committee was formed with expertise in data access, linkage of large health data sets, ethics, health data privacy and legal policy.

## Results

Data governance frameworks and policies from established state registries, large clinical trials and health data sharing and linkage centres (n=7) were reviewed and a summary was presented to the committee. An existing data access and sharing agreement and principles was chosen as a template based on existing stakeholder collaborations and relevance to the two NCHA institutes (Monash University and Peninsula Health). The draft agreement and principles were modified and piloted for data access use cases (n=6). Feedback from researchers (n=3) was used to refine the framework. The committee identified that additional frameworks, such as those outlined by the Centre for Victorian Data Linkages, will be required to accommodate future data sharing and linkage activities with industry and government.

## Conclusion

Our work highlighted the importance of developing a robust governance framework with the ability to incorporate a range of data, that was acceptable to end-users and had sufficient flexibility to incorporate future yet to be identified data types. Ongoing work will expand the framework to include additional data linkage activities.

